# The IASP pain curriculum for undergraduate allied health professionals: educators defining competence level using Dublin descriptors

**DOI:** 10.1186/s12909-020-1978-z

**Published:** 2020-02-28

**Authors:** W. van Lankveld, B. Afram, J. B. Staal, R. van der Sande

**Affiliations:** 1 Musculoskeletal Rehabilitation Research Group, Institute of Health Studies, HAN University of Applied Sciences, Arnhem, The Netherlands; 2 Radboud Institute for Health Sciences, Radboud University Medical Centre, IQ healthcare, Nijmegen, The Netherlands; 3 Faculty of Health and Social Studies, HAN University of Applied Sciences, PO Box 6960, 6503 GL Nijmegen, The Netherlands; 4 Department of Anesthesiology, Pain and Palliative Medicine, Radboud University Medical Centre, Nijmegen, The Netherlands

**Keywords:** Education, Pain curriculum, Undergraduates

## Abstract

**Background:**

Improving pain education for undergraduate health professionals is hampered by lacking shared education outcomes. This study describes how educators and pain experts operationalize content and competency levels deemed necessary for a undergraduate pain education core curriculum for health professionals (physical and occupational therapists, nurses, psychologists).

**Methods:**

Educators and experts on pain and pain education gave their opinion on content and competency level for each individual item of the International Association for the Study of Pain (IASP) inter professional curriculum. Participants decided whether or not to include each item in the undergraduate curriculum. Items were included when > 70% of the respondents agreed. The required competency for each item was rated using ordinal Dublin Descriptors.

**Results:**

Overall, 22 experts rated the curriculum, with > 70% agreement on inclusion on 62% of the IASP items. Within the IASP domain ‘Multidimensional nature of pain’ there was full agreement on the inclusion of 12 items. ‘Ethics’ was considered less important with only 1 item deemed necessary. There is a high number of items selected within the domains ‘Pain Assessment and measurement’ (78%) and ‘Management of Pain’ (74%). Considerably less items were chosen in the domain ‘Clinical Conditions’ (41%). For most items the median required skills and competency level was either Knowledge and Understanding, or Applying Knowledge and Understanding.

**Conclusion:**

Overall, educators and experts in pain agreed on content and competency levels for an undergraduate pain curriculum based on the IASP. Defining a shared competency level will help improve definition of education outcome.

## Background

In Europe, an estimated 20% of the population is suffering from chronic pain [1, 2]. Pain is the most important reason to seek medical attention [3], and poses an important burden for society [4, 5]. As pain is best understood within the framework of the multidimensional biopsychosocial approach different disciplines need to work together in pain management and treatment [6, 7]. In order to make such an interdisciplinary approach successful, a minimum level of shared knowledges and skills is required of disciplines involved [8]. Although pain relief is considered a fundamental human right [9], pain management is inadequate in most parts of the world [10]. Lack of formal education about pain for health professional students might contribute to inadequate pain management [11, 12].

To improve pain education, the International Association for the Study of Pain (IASP) has developed curricula for different health professionals as well as an interprofessional curriculum [13]. The interprofessional curriculum provides a basic overview of suggested topics for interprofessional learning that can be developed further and in more detail uniprofessionally. Such an interprofessional pain curriculum provides a common language as well as a demarcation of major concepts important to all disciplines involved [13]. Of nature, such curricula are likely to be comprehensive, including a vast array of specific pain related topics.

However, when educators try to translate the IASP curriculum into their education program they are faced with a number of problems. Most importantly: the IASP interprofessional curriculum does not describe the content and levels of competency required at an undergraduate level. Most pain education programs described in the literature target postgraduates, with less attention for undergraduate pain education [14, 15]. Undergraduate pain education curricula are often discipline specific [16–18]. Efforts have been made to arrive at a consensus on the content and desired competencies of an interprofessional pain curriculum at a pre-licence or undergraduate level [12, 19]. However, these studies did not use an a-priori defined qualification of competencies shared by all disciplines to describe required competencies for the items in the IASP curriculum. Required competencies for all disciplines have to be clearly defined in order for interprofessional collaboration to be successful.

Several qualifications have been developed to assess competence levels in higher education [20]. Aiming to promote international transparency in higher education qualification, the European Qualification Framework (EQF) describes generic outcome competencies [21, 22]. These generic statements of typical expectations of achievements and abilities associated with qualifications at distinct levels of higher education, can be used to describe required competencies. Such competencies refer to specific capabilities or competency in one specific area [23]. As the IASP interprofessional pain curriculum is comprehensive, encompassing a wide range of pain related items it can be used to determine levels of competency for health professionals pain education at an undergraduate level.

Therefore, the aim of this study is to describe agreement amongst educators and health professionals on content and competency levels deemed necessary for a interprofessional pain education core curriculum at an undergraduate level for Health Professionals (nurses, physical therapists, occupational therapists, and psychologists).

## Methods

In the present study two expert panels were asked to judge the individual items of the IASP interprofessional curriculum on inclusion and required competency level for a interprofessional undergraduate pain curriculum. When exact knowledge regarding a specific topic is not known, researchers have to rely on expert panels to identify and prioritize issues for decision-making [24]. The Delphi method is a popular tool to identify and prioritize decision making. It involves different steps to identify the most important issues of interest by soliciting qualified experts, using controlled opinion feedback. Depending on the research question, different rounds of controlled feedback in an iterative process can be used to generate consensus between experts [24]. For this study we applied just one step op the Delhi study to describe agreement amongst educators and health professionals on content and competence level. The use of a panel of experts is based on the assumption that the pooled knowledge of a group of experts is superior to the individual expert’s knowledge [25]. In panel 1, pain experts involved in an interprofessional pain education programme rated each item. Educators working together in an interprofessional pain education programme make certain choices in developing this programme. It is of interest to see if a panel of international experts could endorse these choices. Therefore, the results of panel 1 were corroborated using panel 2 consisting of a sample of international pain experts not necessarily involved in inter- or multi-disciplinary education.

### Participating experts

Experts in this study were defined as individuals being considered an expert in pain treatment and/or pain education. Following the Delphi approach, a sample of between 10 to 15 experts can yield sufficient results [26], assuring validity [27]. In this study we aimed to include the recommended minimum sample size of 20 [24]. Panel 1 consisted of experts participating as educator in a bachelor interprofessional training program in pain and pain management at the HAN university of applied sciences in Nijmegen, The Netherlands. All educators had extensive experience working in an interdisciplinary setting. The interprofessional training is open for students in four disciplines that most frequently work together in pain treatment in the Netherlands (nursing, physical and occupational therapy, and psychology). A total of 7 experts contributed to the development and delivery of the content of the programme requiring intense discussion between participating disciplines about the required levels of education at such an interprofessional curriculum. For panel 2, international experts on pain and pain education were contacted with the request to participate in the study. The following groups of experts were considered for inclusion in panel 2: 1) Pain educators with a Health Care Professional backgrounds (for instance medical doctors, physical therapists, occupational therapists, nurses, psychologists, etc.) 2) Pain professionals participating in health professional’s education (both acute and chronic pain). 3) Pain researchers if they have a connection with undergraduate professional teaching. 4) Researchers in didactics and development of pain education. Furthermore, we sought an equal mix of experts from different professions. The desired number of experts was set at 3 for each discipline. International experts for the second panel were recruited using the following means: 1) IASP curriculum members were contacted through the chair members of the curriculum committee of each health profession. 2) Poster presentation during EFIC and IASP conferences. Attendees who were interested, and who fulfilled the requirements were invited to participate; 3) the networks of already participating experts were used to further recruit experts.

### Measurement

The IASP interprofessional curriculum consists of 107 items deemed important for health professionals working with patients with pain [13]. The curriculum is divided in 4 main domains: I. Multidimensional nature of pain (18 items); II. Pain Assessment and measurement (18 items); III. Management of Pain (35 items) and IV. Clinical conditions (36 items). Within each domain items are grouped into themes. The current online Interprofessional Curriculum has been updated since performing this study. The organisational structure of the curriculum has largely been kept intact, but the items have been numbered otherwise, and within some themes the number of items has been extended. To allow the readers to compare the results to the current online version, the numbering of the online version is used. Furthermore, for each domain the difference in items included in the updated curriculum compared with the old curriculum will be described.

Participants in panel 1 were presented with the individual IASP items on paper. For each item the expert was asked to rate whether or not this item should be included in an undergraduate interprofessional pain curriculum. If an item was included, the expert was asked to rate the level of competency required using the Dublin Descriptors using a 5 point scale [21]. The meaning of the five successive, and hierarchical levels are presented in Table 1. It was emphasized that it regards a required core curriculum, and that each profession or institution can decide whether additional topics or a higher level of competency is necessary for their particular students.

**Table 1:** Dublin Descriptors

Level number	Level title	Description on Bachelor level
1	FYI (for your information)	The topic is only mentioned for information. No further action or skill is requested from the student
2	Knowledge and understanding	Student shows knowledge and understanding in a field of study at a level that, whilst supported by advanced textbooks, includes some aspects that will be informed by knowledge of the forefront of their field of study
3	Applying knowledge and understanding.	Student can apply their knowledge and understanding in a manner that indicates a professional approach to their work or vocation, and have competences typically demonstrated through devising and sustaining arguments and solving problems within their field of study
4	Making Judgements	Student has the ability to gather and interpret relevant data (usually within their field of study) to inform judgements that include reflection on relevant social, scientific or ethical issues
5	Communication	Student can communicate information, ideas, problems and solutions to both specialist and non-specialist audiences

Next, the international experts in panel 2 were presented with each item of the IASP interprofessional curriculum using a web-based questionnaire. Together with the invitation to complete the web-based questionnaire they received a description of the Dublin descriptors.

First, the experts in panel 2 were asked to rate whether each item should be included at the undergraduate inter professional level. At this stage, the experts in the second panel were kept unaware of the results of panel 1. Only when the expert in panel 2 rated the item to be included in the curriculum, the web-based questionnaire automatically presented the median level of competency as suggested by panel 1. The expert in panel 2 than had to decide whether to corroborate the suggested level. If the expert in panel 2 did not agree, an additional question was posed to indicate the suggested level of competency for that item.

### Analysis

The results will be described for each item aligned in accordance with the four main domains of the IASP interprofessional curriculum. For both panels, results are given for both groups of experts separate, as well as for all experts combined. In this way, agreement between the lecturers participating in an interprofessional education program (panel 1), and the international experts (panel 2) is described. For each item the percentage of respondents in favor of inclusion in a core curriculum is given, and the median score of required level of competency.

First, for each item data are provided related to consensus of inclusion of that item in the curriculum. Acceptable levels of agreement in Delphi studies might vary depending on the topic, but a level of agreement of 70% is frequently used [28] and was adopted for this study. When more than 70% of the experts is in favour of including an item in the curriculum this was considered agreement between experts. For both the experts in panel 1 and panel 2, and for all experts together, the percentage of participants in favour of including that item in the curriculum is calculated. When in both groups of respondents more than 70% is in favour of an item, there was agreement on inclusion of the item. Similarly, when both groups rated less than 70% the item there was agreement to exclude the item. When both groups were in disagreement (one of the groups lower than 70% and one group higher than 70%) the overall percentage was considered for in- or exclusion.

Next, for each of the items deemed necessary for undergraduate education, a second analysis was performed to decide the level of competency of that item based on the scoring of Dublin Descriptors. Required level of competency for each item was determined first by looking at the median score suggested by those respondents in panel 1 who favoured inclusion of a particular item in the curriculum. Since the levels are hierarchical, a higher level requires competency on all previous levels. For example, in order to apply knowledge and understanding (level 3), one should also have the knowledge and understanding (level 2). When the expert in panel 2 included the item in the curriculum, the suggested median score on level of competency of panel 1 was presented to panel 2 for corroboration or rejection. If the experts in panel 2 corroborated the level of competency of panel 1 there was full agreement, if panel 2 did not corroborated the level of competency the median score was calculated for all respondent in panel 1 and 2.

## Results

A total of 22 experts in two panels were consulted to determine the content of the pain curriculum. Descriptive data about the participants are presented in Table 2.

**Table 2:** Descriptives of respondents

	Panel 1. Lecturers (*N* = 7)	Panel 2.International experts (*N* = 15)
Gender % (*n*)		
Male	57,1% (4)	33.3% (5)
Female	42.9% (3)	66.7% (10)
Profession % (*n*)		
Medical Docter	14.3% (1)	20% (3)
Nursing	28.5% (2)	13.3% (2)
psychology	14.3% (1)	20% (3)
Occupational therapy	14.3% (1)	13.3% (2)
Physical therapy	28.5% (2)	33.3% (5)

All seven lecturers involved in the inter professional pain education programme among allied health professions participated in panel 1. A total of 35 international pain experts were contacted to participate in panel 2 of which 15 agreed to participate. International experts participating in panel 2 originated and worked in the following countries: Australia; Belgium; Canada; Germany; Kenya; Spain (all *n* = 1); Sweden; Switzerland (both *n* = 2); the Netherlands (*n* = 3) and the UK (n = 2).

### Domain I: multidimensional nature of pain

Table 3 depicts the 18 items composing the domain multidimensional nature of pain. The items in this domain are grouped in four themes: *Epidemiology, (development of) pain theories, Mechanisms, and Ethics.* For both panels the percentage of respondents in favor of inclusion of that item in the curriculum is given, as well as the median score in Dublin Descriptors (DD).

**Table 3:** Multidimensional Nature of Pain: percentage of participants in panel 1 and 2 in favor of including the item, and median Dublin Descriptor

	% in favor for inclusion			Dublin Descriptor		
	Panel 1	Panel 2	Total	Panel 1	Panel 2	comment
A.Epidemiology						
1. Pain as a public health problem with social, ethical, legal and economic consequences.	100	93	95	3	3	
2. Epidemiology with overview of statistics related to acute, recurrent and/or persistent (chronic) and cancer pain.	100	93	95	2	2	
3. Barriers to effective pain assessment and management.	100	93	95	3	3	
B. Development of pain theories						
1. Historical development of pain theories and basis for current understanding of pain.	71	93	86	2	2	
2. Definition of pain and pain terms.	100	100	100	3	3	
3. Classification systems of pain.	100	87	91	2	2	
4. Differences between nociception, pain, suffering and harm.	100	100	100	3	3	
5. Pain and behavior.	100	93	95	3	3	
C. Mechanisms						
1. Anatomy and physiology to include neural mechanisms.	100	93	95	2	2	
2. Multiple dimensions of pain.	100	93	95	2	2	
3. Pathological consequences of unrelieved pain, and implications of being a multidimensional experience.	100	100	100	2	2	
4. Factors influencing neurophysiology.	71	66	68			excluded
D. Ethics						
1. Ethical standards of care (provision of measures to minimize pain and suffering) for health care professionals.	71	93	86	2	2	
2. Ethical standards and guidelines related to use of analgesics.	43	73	64			excluded
3. Inadequate pain management for specific groups.	57	73	68			excluded
4.Legal issues related to disability, compensation	29	20	23			excluded
5. Political and societal issues related to access to pain management and attitudes to marginalized populations.	29	42	41			excluded
6. Experimental pain issues related to appropriate and meaningful measures and methods.	57	40	45			excluded

Dublin Descriptors: 1) for your information); 2) Knowledge and understanding; 3) Applying knowledge and understanding; 4) Making Judgements; 5) Communication

Updated version: The updated version of this domain in unchanged

For 12 of the 18 items (66.6%), the threshold of 70% to include the item was reached in both panels. Table 3 shows that all items within the themes *Epidemiology* and *(Development of) Pain Theories* were endorsed in both panels. Within the theme *Mechanisms of Pain* there is agreement on three of the four items. The item *Factors influencing neurophysiology* did not reach 70% agreement in both panels. The items included for the first three themes showed a strong consensus (> 90%). Within the theme *Ethical standards,* there was agreement between panels to include 1 of 6 items: Ethical standards of care (provision of measures to minimize pain and suffering) for health care professionals. Furthermore, there is agreement between both panels that both legal and political issues can be excluded. Within the theme “Ethics”, there was a discrepancy between the panels as whether to include item D.2 and D.3. Both items were excluded based on the overall percentage (< 70%). Next, the median score in Dublin Descriptors depicting required level of competency was considered. In all of the 12 items included, the level of competency suggested by panel 1 was endorsed by panel 2. For 7 items the median score in both panels was 2 (knowledge and understanding), for 5 items the median required level was 3 (applying knowledge and understanding).

### Domain II: pain assessment and measurement

This domain includes 18 items grouped in three themes: *Interprofessional and Multiprofessional collaboration, Assessment,* and *Measurement.* The specific results of all items can be found in Table 4.

**Table 4:** Pain assessment and Measurement: percentage of participants in panel 1 and 2 in favor of including the item, and median Dublin Descriptor

	% in favor for inclusion			Dublin Descriptors		comment
	Panel 1	Panel 2	Total	Panel 1	Panel 2	
A. Interprofessional and Multiprofessional collaboration						
1. Comprehensive assessment.	100	93	95	3	3	
2. Clear documentation of pain assessment and measurement data	100	93	95	3	3	
3. Ongoing communication for comprehensive and consistent approaches	100	73	82	3	3	
4. Monitoring of efficacy and effectiveness of management plan	100	87	82	3	3	
5. Consideration of appropriate assessment and measurement approaches for people with special conditions	86	71	76	2	2	
6. Development of interprofessional consultant networks (informal/formal) when needed for adequate assessment with complex patients	86	57	67			excluded
B. Assessment						
1. History: Pain location, onset and duration, severity, quality, alleviating and aggravating factors; Impact on mood, usual activities/function/quality of life/sleep	100	93	95	2	2	
1. c. History: Previous pain and treatment history.	100	93	95	2	2	
1.d.History: Ongoing response to treatment, adverse effects	100	86	90	2	2	
1.e.History: Comorbidities impacting pain	100	93	95	2	2	
1.f-.History: Personal characteristics	86	86	86	2	2	
1.g.History: Expectations of pain management and current understanding of the condition	100	93	95	2	2	
2. Physical examination	71	50	57			excluded
3. Review of clinical records	57	71	67			excluded
4. Investigations (Laboratory tests and Imaging)	43	29	33			excluded
C. Measurement						
1. Approaches (Qualitative and Quantitative)	100	86	90	2	2	
2. Testing issues	100	79	86	2	2	
3. Tools (uni- and multi-dimensional)	100	93	95	3	3	

Dublin Descriptors: 1) for your information); 2) Knowledge and understanding; 3) Applying knowledge and understanding; 4) Making Judgements; 5) Communication

Updated version: In the updated version the following themes were further divided in different items: B.2., B.3, B.4, C.1, C.2, C. 3

For 14 of the 18 items (78%), the 70% threshold for inclusion was reached by both panels. Within the theme *Interprofessional and multiprofessional collaboration* all but 1 item were included. The item “Development of inter professional consultant networks (informal/formal) when needed for adequate assessment with complex patients” was considered relevant by the panel 1 (86%), but less so by panel 2 (57%). As the overall percentage was lower than 70%, this item was excluded. Within the theme *Assessment* 6 items were included and 3 items were excluded. Both panels agreed to exclude the item Investigations. On two items in this theme (Physical exam, and Review of Clinical Records) the panels did not agree. As these items had less than 70% cumulated agreement for inclusion, they were excluded. Within the theme *Measurement*, all three items were included by both panels.

Considering the levels of competency deemed necessary at an undergraduate level, the second panel endorsed the level of competency as proposed by the first panel. The Dublin Descriptors attached to the individual items most often referred to knowledge and understanding (Dublin Descriptors level 2), and some to applying knowledge and understanding (level 3). With the exception of 1 item, all items related to the theme Interprofessional and Multiprofessional collaboration should be performed at level 3.

### Domain III: Management of Pain

The domain Management of Pain includes 35 items grouped in 15 themes. Items and scores are depicted in Table 5.

**Table 5:** Management of Pain: percentage of participants in panel 1 and 2 in favor of including the item, and median Dublin Descriptor

	% in favor for inclusion			Dublin Descriptors		
	Panel 1	Panel 2	Total	Panel 1	Panel 2	Comment
A.Goals of Pain Management.						
1. Prevention and/or reduction of pain intensity.	86	93	90	3	3	
2. Enhancement of physical functioning.	100	93	95	3	3	
3. Improvement of psychological functioning.	100	93	95	2	2	
4. Promotion of return to work/school and/or role within the family/society.	100	93	95	3	3	
5. Improvement of health-related quality of life.	100	93	95	3	3	
B. Pain Management Planning Decision.						
1. Develop, monitor and modify the management plan as an interprofessional and/or multiprofessional team.	100	79	86	2	2	
2. Involve patient and family caregivers in establishing clear, realistic goals.	100	93	95	3	3	
3. Use combinations of methods where appropriate including physical, psychological, pharmacological and interventional.	100	86	90	2	2	
4. Provide patient information/education including: communication methods, management options, management of potential adverse effects.	100	100	100	3	3	
5. Develop transparent treatment plan with realistic goals.	100	79	80	3	3	
C. Treatment Considerations.						
1. Type(s) of pain.	100	86	90	2	2	
2. Multidimensional nature of pain (e.g. Biological, psychological, social) Use of combinations of pharmacological and non-pharmacological methods.	100	100	100	3	3	
D. Patient issues.	86	86	86	2	2	
E. Caregiver issues.	86	86	86	2	2	
F. Health professional issues.	86	93	90	2	2	
G. Political issues.	71	79	76	1	1	
H. Substance use disorders / abuse issues.	57	64	60			excluded
I. Consider Non and use non-pharmalocial /interventional stategies (not in old version).						
J. Pharmacological Methods.	57	43	48			excluded
K. Clarify tolerance, physical dependence and psychological dependence.	29	79	62			excluded
L. Utilize combinations of analgesics and adjuvants where appropriate.	29	43	38			excluded
M. Knowledge of legislative requirements and current guidelines regarding controlled drugs.	29	21	24	–		excluded
N. Non-pharmacological and intervention methods.						
1. a. Clinician therapeutic use of self .	100	93	95	3	3	
1. b. Physical strategies to support home and occupational function and activity.	100	86	90	3	3	
1.c.Psychological and behavioral strategies	100	100	100	3	3	
1.d.I.Neuromodulation	71	79	76	2	2	
1.d.II.Neuroablative strategies	71	50	57			excluded
1.d.III.Procedural/Interventional	71	57	62			excluded
1.d.IV.Surgery	86	50	62			excluded
1.d.v. Palliative Radiotherapy	71	50	57			excluded
2.Complementary alternative medicine (CAM)	57	46	57			excluded
3. Information and communication technologies	100	57	71	3	3	
O. Evaluation of outcome						
1. Monitor management outcomes .	86	79	81	3	3	
2.Utilize an interprofessional and multiprofessional team approach	71	93	86	2	2	
3.Consider barriers related to treatment availability and costs at the patient-family, institution, society and government levels	57	57	57			excluded

Dublin Descriptors: 1) for your information); 2) Knowledge and understanding; 3) Applying knowledge and understanding; 4) Making Judgements; 5) Communication

Updated version: In the updated version 1 item has been dropped from theme 1 (Reduction of healthcare utilization), and 1 theme has been added (Theme I). Furthermore, the following themes were further divided in different items: D to J

Overall, the experts included 26 out of the 34 items in this domain (76%). There was agreement between both panels to include all items within the themes *Goals of Pain Management, Pain Management Planning Decisions, Treatment Considerations, Patient Issues, Caregiver Burden,* and *Health Professional Issues.* For these themes, each item was selected by a large majority of respondents (80–100%). The Theme *Political Issues* was selected by only 76% of the participants. Themes H to M were excluded. These themes did not reach the 70% cut-off score in both panels, or did not reach the cumulated score of 70%. Within the theme *Non-pharmacological and Intervention Methods,* there is a large differences in rating of items. Three items (Clinical Therapeutic Use of Self, Physical strategies to support function and activity, and psychological and behavioral strategies) are supported by nearly all respondents, while neuromodulation just reached the 70% threshold in both panels. Among all other items of the theme *Treatment Consideration* one of the response groups did not meet the 70% cut-of score. The item *Information and communication technologies* was included because it was selected by more than 70% of all the respondents combined. Neuroablative strategies, Surgery, CAM and Palliative radiotherapy, all failed to meet the cumulated 70% cut off score. Finally, within the theme *Evaluation of Treatment Outcome* both groups of respondents were in agreement about all items; two items were selected by both groups of respondents, and one item was excluded.

In the 26 items that were deemed necessary to be included in the curriculum, the required level of competency indicated by panel 1 was corroborated by panel 2. The required level of competency assessed indicates that health professionals should, at an undergraduate level, know and understand the selected items (level 2), and for many items to be able to apply this knowledge (level 3). Only the item Political Issues is rated as level 1: mentioned for information.

### Domain IV: clinical conditions

This final domain contains 37 items grouped in eight themes: *Taxonomy of pain systems, Pain in special populations, Acute time-limited pain, Cancer pain, Visceral pain, Headache and Facial Pain, Neuropathic pain, and Musculoskeletal.* Table 6 gives information about the individual items.

**Table 6:** Clinical Conditions: percentage of participants in panel 1 and 2 in favor including the item, and median Dublin Descriptor

	% in favor for inclusion			Dublin Descriptors		comment
	Panel 1	Panel 2	Total	Panel 1	Panel 2	
A.Taxonomy of Pain Systems.						
1. Distinction between acute, recurrent, incident, and or persistent (chronic) pain.	100	92	95	3	3	
2. Distinction between nociceptive (somatic, visceral) and non-nociceptive (neuropathic) pain.	100	92	95	3	3	
3. Distinction between commonly used pain terms in clinical practice.	86	100	95	3	3	
4. Involvement of biological, psychological and social factors influencing the perception of pain.	100	92	95	3	3	
B. Pain in special populations						
1. Pain in infants, children and adolescents.	43	79	67			excluded
2. Pain in older adults.	86	73	76	2	1	DD difference
3. Pain in individuals with limited ability to communicate.	29	71	57			excluded
4. Pain in pregnancy, labor, breast feeding.	29	36	33			excluded
5. Pain with psychiatric disorders.	14	50	38			excluded
6. Pain in individuals with substance abuse.	29	43	38			excluded
C: acute time-limited pain.						
1. Surgery	86	64	71	1	1	
2. Trauma	71	71	71	2	2	
3. Infection	71	57	62	2	2	excluded
4. Inflammation	86	86	86	1	1	
5. Burn	43	43	43	–	–	excluded
*D. cancer* pain						
1. Primary pain	57	71	67			excluded
2. Local invasion	57	43	48			excluded
3. Metastatic spread	57	50	52			excluded
4. Treatment-related	57	71	67			excluded
5. End-of-life	43	71	62			excluded
E. Visceral pain						
1. Referred patterns	43	64	57			excluded
2. Cardiac and non-cardiac chest pain	43	36	38			excluded
3. Abdominal, peritoneal, retroperitoneal pain	29	21	24			excluded
4. Pelvic pain (male and female)	29	50	43			excluded
5. Sickle cell crisis	14	21	19			excluded
F. Headache and Facial Pain						
1. Headache	43	71	62			excluded
2. Orofacial pain	43	57	52			excluded
3. Trigeminal neuralgia	29	57	48			excluded
G. Neuropatic Pain						
1. Primary Lesion Central.	71	71	71	2	2	
2. Primary Lesion Peripheral.	71	86	81	3	3	
3. Mixed or unclear origin.	100	86	90	1	3	DD differs
H. Musculoskelethal Pain.						
1. Rheumatoid arthritis, osteoarthritis.	100	71	81	2	2	
2. Neck pain, whiplash and referred pain.	100	71	81	2	2	
3. Low back pain and referred pain.	100	93	95	2	2	
4. Injuries from athletics.	86	57	67			excluded
5. Myofascial pain syndrome.	86	57	67			excluded

Dublin Descriptors: 1) for your information); 2) Knowledge and understanding; 3) Applying knowledge and understanding; 4) Making Judgements; 5) Communication

Updated version: The updated version of this domain in unchanged

Table 6 shows that 16 items in this domain were included (41%), indicating that 21 out of 37 items in this domain failed to reach the 70% overall limit to be included in an undergraduate inter professional curriculum. All items in the themes *Taxonomy of Pain Systems* (4 items) and *Neuropatic Pain* (3 items) meet the 70% cut-off criterion in both panels separately. Within the theme Taxonomy there is nearly full endorsement (> 90%). Within the Theme Pain in special Populations, pain in the elderly was the only item included. All other items in this theme were excluded either because both panels agreed to excluded this item (Pain in Pregnancy, Pain in Psychiatric disorders, and Pain in individuals with Substance Abuse), or panels differed on inclusion, and exclusion was based on the cumulated score (Pain in Infants, Pain in Individuals with limited ability to communicate). Within the theme *Acute time limited pain*, trauma and inflammation were selected by both groups, and both panels agreed to exclude Burns. The item surgery was included based on the cumulated percentage in favor of inclusion. Within the theme *Musculoskeletal* 3 of the 5 items met the 70% criterion, with low back pain and referred pain reaching almost full endorsement by all participants. Items related to Cancer Pain, Visceral pain, and Headache and Facial Pain did not meet the 70% criterion in both groups.

When considering the levels of competency deemed necessary for undergraduate student, both panels were in agreement on most of the items included. Taxonomy and Pain systems is required at the application of knowledge (level 3). There was disagreement in two items on the level of competency required: The item Pain in Older Adults was suggested by panel 1 at level 2 (know and understand the selected items), but panel 2 did not agree and thought it sufficient when it is mentioned for information (level 1). The item Neuropatic Pain of mixed of unclear origin, was suggested by panel 1 at level 1, whereas panel 2 suggested that students must be able to apply this knowledge (level 3).

## Discussion

This is the first study that describes levels of consensus on required competency levels for a core curriculum for health professionals at an undergraduate level from the educators point of view. Based on the expert opinion, 60% (64 of the 107 items) of the IASP interprofessional curriculum items should be included in a undergraduate health professional pain curriculum. The percentage of items included for the domains Multidimensional Nature of Pain, Pain Assessment and Measurement, and Management of Pain was respectively 66, 78, and 74%. In the domain Clinical conditions only 41% of the items were chosen by the experts. Some themes of the IASP curriculum are included in their entirety with high inclusion rates by the experts (> 85%). These are: Epidemiology, (development of) Pain Theories and Mechanisms within the domain Multidimensional nature of pain; Measurement within the domain Pain assessment; Goals of Pain Management, Treatment Considerations, Patient Issues, Caregiver Burden, and Health Professional Issues in the domain Management of Pain, and Taxonomy of Pain Systems in the domain Clinical Conditions. These themes and their items are generally considered important to all health professionals and should preferably be incorporated in the core curriculum. In other themes, a smaller number of items was selected for inclusion. For the majority of items both panels of experts were in agreement: for 61 items (57%) both panels were in favor for inclusion, and for 24 items (22%) both panels decided to excluded the item. Only for 22 items (21%) there was a difference between both panels. As to the level of competency required at a health professional undergraduate level, the level of competency suggested by panel 1 was endorsed by panel 2 in all but two items. For most items the median level of competency assessed using Dublin Descriptors was either 2 (Knowledge and Understanding) or 3 (Applying Knowledge and Understanding).

Most of the 39 items that were excluded based on the expert opinion belong to the Domain Clinical Conditions. Experts were asked to select only those items they considered mandatory for all disciplines involved. Because not all health professionals will be confronted with all these conditions (Cancer Pain, Visceral pain Headache and Facial Pain), it might have been expected that not all items are deemed important for all health professionals at an undergraduate level. On some items there is a large difference in the panel’s votes. For instance, the item ‘development of interprofessional consultant network’, is selected by 86% in panel 1, against 57% in panel 2. This difference might be the result of the way both panels were selected, as the participants of panel 1 work together in the same interprofessional pain program. Differences between both panels on other items is less easy to understand. Within the theme *Special population* of the Domain Clinical Conditions the only item included was Pain in Older Adults. Pain in Infants was excluded although 79% of the international experts were in favor of inclusion. Overall, this study showed that on some themes of the IASP interprofessional curriculum there is full agreement amongst all experts both on the content and the competency level of the items in the curriculum. For the overwhelming majority of items, both panels of experts (panel 1: interprofessional teachers, and panel 2: international experts) were in agreement to include or exclude any particular item. The content of items included in this study shows large overlap with curriculum content described in earlier studies [12, 19]. However, the required competency of each of the IASP items sets this study apart from these earlier studies aiming to identify core competencies for pain management at an undergraduate level. The level of competency required frequently differed for individual IASP items within one theme, but for 62 of the 64 items included there was agreement between both panels about the required competency at an undergraduate level.

This study is not without its limitations. The validity and reliability of an expert panel’s judgement is highly dependent on the expert selection. For this study we recruited and included experts with both expertise in pain management and pain education. These requirements were not controlled for, and study participation relied on the experts own assessment of these requirements. As a result it is not known whether the expert panel is relative homogeneous [26] and validity cannot be assured [27]. However, the high concordance between both panels assessments of items included in the curriculum suggests high reliability and validity. Another limitation related to the choice of experts is the domination of experts from western, highly developed countries. It is unclear how this bias in expert selection is reflected in the choices made, and whether these choices can be generalized to non-western countries. A methodological limitation of the study is the evaluation of items using the Dublin Descriptors. These Descriptors are very broadly defined and lacking in operationalisation. Therefore, there is ample room for different interpretation between experts. Furthermore, in this study we used a two step approach to arrive at a suggested Dublin Descriptors level. Experts in panel 2 were presented with the suggested level of competency of panel 1. This was done because it was expected that most international experts had no previous experience working with Dublin Descriptors. However, prompting panel 2 with the suggested level of competency based might present a bias. A final limitation of this study to be mentioned is the fact that the expert opinion was asked to rate the items of the 2012 curriculum, recently updated in 2017. The updated version does not differ from the original in overall structure, but differs in the detailing of subdividing items. However, as the updated version is largely consistent with the previous version, the differences are limited.

Nevertheless, this study is helpful to define required competencies for all disciplines on pain education for health professionals at an undergraduate level. As stated in the IASP curriculum, this outline provides a basic overview of suggested topics for interprofessional learning. It does not replace the uniprofessional curricula that provide additional depth in content required by each individual profession and discipline. For instance, for disciplines working with patients and substance abuse disorders additional information is needed. The method described in this study might help other professionals considering how to take a curriculum designed for one audience and to modify it for another.

However, defining the boundaries of a pain curriculum for undergraduate health professionals is only a first step in improving pain education. As pain is best approached using interdisciplinary collaboration, it has been argued that students should be enabled to learn about pain using Inter Professional Education (IPE) [7, 29]. IPE occurs when members of more than one profession interactively learn together, for the explicit purpose of improving interprofessional collaboration or the health/wellbeing of patients/clients (or both) [30]. Although the merit of IPE has been recognized [30–32], the incorporation of IPE in the health professionals’ education is limited due to a large number of professional, institutional, and individual barriers [33]. Enablers of IPE education include unified goals and shared interprofessional vision [33]. Therefore, IPE pain education for AHP could benefit from an operationalization of interdisciplinary pain curriculum at the undergraduate level. A key recommendation from the 2009 survey on undergraduate pain curricula in the UK is: Encourage opportunities for interprofessional pain education, in the undergraduate curriculum, to mirror practice and promote understanding of individual roles [11].

## Conclusion

Interprofessional collaboration in pain treatment is conditional upon shared levels of knowledge and capabilities. Defining a shared competency level at an undergraduate level will help improve definition of education outcome. Overall, educators and experts in pain agreed on content and competency levels for an undergraduate pain curriculum based on the IASP. This study is helpful to define required competencies for all disciplines on pain education for health professionals at an undergraduate level.

## Data Availability

The data that support the findings of this study are available from HAN University of Applied Sciences but restrictions apply to the availability of these data, which were used under license for the current study, and so are not publicly available. Data are however available from the authors upon reasonable request and with permission of HAN University of Applied Sciences.

## Abbreviations

AHP: Allied Health Professionals
DD: Dublin Descriptors
EQF: European Qualification Framework
FYE: For Your Information
IASP: International Association for the Study of Pain
IPE: Inter Professional Education
